# Acknowledgment to the Reviewers of *Behavioral Sciences* in 2022

**DOI:** 10.3390/bs13020107

**Published:** 2023-01-28

**Authors:** Behavioral Sciences Editorial Office

**Affiliations:** MDPI AG, St. Alban-Anlage 66, 4052 Basel, Switzerland

High-quality academic publishing is built on rigorous peer review. *Behavioral Sciences* was able to uphold its high standards for published papers due to the outstanding efforts of our reviewers. Thanks to the efforts of our reviewers in 2022, the median time to first decision was 18 days and the median time to publication was 41 days. Regardless of whether the articles they examined were ultimately published, the editors would like to express their appreciation and thank the following reviewers for the time and dedication that they have shown *Behavioral Sciences*:

Aan KomariahKayvan AghabaykAaron ManzanaresKe ShaoAaron ShieldKen MastersAaron ZimmermanKensaku TakaseAbbas MardaniKeonya BookerAbdulaziz AlsulamiKevin KantonoAbel LermaKevin Matthe CaramancionAbhishek KaushikKimberly A. HoffmannAbraham RudnickKimberly WingertAbu Al Nasr SobaihKinga KimicAbu Baker SheikhKirill MiroshnikAdam FalewiczKiyotaka NemotoAdam NovotnyKlára ČermákováAdam ReevesKleftodimos AlexandrosAdam SafronKourken MichaelianAdamides EmmanuelKristina MarintsevaAdela BadauKrystyna Mazurek-ŁopacinskaAdil MansoorKrzysztof JurekAdil ZiaKrzysztof ZdziarskiAdnan Ul-HaqueKsenija MarinkovicAdonis HaidarKudret TopyanAdrian StancuKumpei MizunoAgata SudolskaKun GaoAglaja StirnKun-Hua LeeAgnieszka BojanowskaKyle MoodyAgnieszka Knap-StefaniukKyle S. MinorAgnieszka PlutaKyunghwa ChoAgnieszka TruszkowskaKyung-Hyun SuhAgus SugiartoLadislav StankeAgustín Llopis-GonzálezLajuan DavisAhmed Hassan Hemdan MohamedLana MinshewAhmed Mohamed ElbazLara Orcos PalmaAidin SalamzadehLászló VasaÁine Ní ChoisdealbhaLászló VécseiAkira MinouraLaura Elena MarinașAlba Viana LoraLaura Fusar-PoliAlberto Frutos Pérez-SurioLaura GuerreroAlejandro Martínez-RodriguezLaura Lucas-PalaciosAlejandro Rabasa DoladoLaura NathansAleksandra DraginLaura QuirosAlena A. ZolotarevaLaura Redondo GutiérrezAlessandra CostanzaLaura SalernoAlessandro Lo PrestiLeeanne Bell McManusAlessia GrigolettoLeonel PrietoAlexander JedingerLesley NewsonAlexandre González-RodríguezLetícia Seixas PereiraAlexandru TibaLia AraujoAlexios BatrakoulisLidia GavicAlfonso Conde LacárcelLiliya PoskotinovaAlfonso Martínez-MorenoLinda KuranAlfonso Meneses MonroyLindsey Conlin MaxwellAli BodlaLinus Mabughi NyiwulAlice LemmoLionel Sanchez-BolivarAlicia Orea-GinerLisa HaleAlim SyariatiLiu YiAlina BetlejLiubov TkachevaAlina CernasevLiza LeeAlison AndersonLizandra Garcia Lupi VergaraAlison R. CoelhoLjubica KaziAlla Alla PakinaLluís AlbarracínAlma EspartinezLorenzo ViselliAltti NäsiLoreta Buksnyte–MarmieneÁlvaro Antón-SanchoLoreta CannitoÁlvaro Díez RevueltaLori GrayAlyona IvanovaLourdes Chocarro GonzálezAlyson J. ChroustLovorka BrajkovićAlyx TaylorLua Perimal-LewisAmaia YurrebasoLuca J. SteardoAmalia CorneaLuca PetrignaAmanda SorensenLuca SimioneAmaya Epelde-LarrañagaLucia RatiuAmelia Beata StaszowskaLuciano Ferreira Da SilvaAmélia SilvaLufanna Ching-han LaiAmelia V. García-LuengoLuigia Simona SicaAmir GhanbaripourLuis Carrasco PáezAmjad KhanLuis CarroAmrut SadacharLuís Felipe Dias LopesAmy M. SchmisseurLuiz Felipe SilvaAna AleksićLuka PavličAna Ancheta ArrabalŁukasz TomczykAna Cocho-BermejoLuminita NicolescuAna Junça-SilvaLuz Adriana Aristizábal BecerraAna Manzano-LeónLydia Giménez-LlortAna María Botella NicolásLyndon LimAna María Martín RodríguezLynn M. PezzaniteAna Maria Mihaela IordacheM. A. MasudAna María Ruiz-Ruano GarcíaMacarena Castellary LópezAna Martínez-PampliegaMaciej DębskiAna Paula Camargo LaroccaMaciej KochmanAna Paula MonteiroMaciej SerowaniecAna Rodríguez-MartínezMack ShelleyAna SabinoMadelon L. FinkelAna SaniMagdalena Anna JaworekAna VazquezMagdalena BłażekAna ŽivkovićMagdalena IorgaAnamaria CiubaraMagdalena Mackiewicz-MilewskaAnanya RajagopalMagdalena Ramos Navas-ParejoAnastasia BauerMahfuzur RahmanAnastasia MiklyaevaMai Salah El Din Mohamed HelmyAnca Antoaneta VarzaruMaja RožmanAnca BobircaMajid Mufaqam Syed-AbdulAnca-Maria ClipaMałgorzata Anna BasińskaAndré L. L. BachiMałgorzata MacudaAndrea BalliniMałgorzata NagórskaAndrea BoscoMałgorzata OkręglickaAndrea MeluchMalgorzata RutkowskaAndrea PompignaMałgorzata StachońAndreas AltorferMałgorzata ZakrzewskaAndreas ElpidorouManfred ZentnerAndreas JacobssonMansoor SoomroAndree HartantoManuel Luís CapelasAndreea-Loreta CercleuxManuel PereiraAndrei HolmanMarawan AbdelBasetAndrei TernauciucMarc Perez-BurrielAndreia LimaMarcin KomandaAndrés Barrios-RubioMarcin PasekAndrés Calvillo-TéllezMarcin SiwekAndrés Fernando Avilés-DávilaMárcio OliveiraAndrey D. NasledovMarco BertaminiAndrzej AdamskiMarco CambiaghiAndrzej BąkMarco De AngelisAndrzej PiotrowskiMarco GiannelliAneta GrochowskaMarcosiris Amorim De Oliveira PessoaAngel De JuanasMarek AngowskiAngela FinkMarek BodzianyAngela HewettMarek KruszewskiAngela RepanoviciMargaret Bennett-BrownAngelika Tkaczyk-WlizłoMargaret LeahyAngelo Di IorioMargareth Santos ZanchettaAngelo Mark P. WalagMargo TurnbullAni GasparyanMaría Antonia Parra-RizoAnik YuestiMaria Ceu MacHadoAnna Barwińska-MałajowiczMaria Chiara CascheraAnna CybulskaMaría Cristina Martínez-FernándezAnna DodonovaMaria Da Conceição TavaresAnna DziadkiewiczMaría Del Carmen García-MendozaAnna IrimiasMaría Del Mar Fernández-MartínezAnna KamzaMaría Del Mar Sánchez RamosAnna Mazurek-KusiakMaría Dolores Díaz NogueraAnna MnatsakanovaMaria GagarinaAnna ParolaMaria GrajdianAnna Rogozińska-PawełczykMaria Grazia TarsitanoAnna SaitiMaría Jesús HerreroAnna StankowskaMaria LucaAnna Strychalska-RudzewiczMaría Luisa BelmonteAnna TychmanowiczMaria Luisa IndianaAnna WłochMaría Magán-MagantoAnna ZalewskaMaria Margarida Croca PiteiraAnnamaria NeseMaría MarínAnne RoskenMaria MartzoukouAntonella PomeMaria NathanAntonino ManiaciMaría Orosia Lucha-LópezAntonio BovaMaria Raquel BarbosaAntónio CardosoMaria Rella RiccardiAntonio Jesús Molina FernándezMaria SarapultsevaAntonio Martínez RayaMaria TatarusanuAntonio TintoriMaria Teresa GiglioAntonio-Juan Briones-PeñalverMaría Yolanda Castellote-CaballeroAntum A. PanjwaniMaria-Crina RaduAnupa Ambili VijayakumariMaría-Leticia Meseguer-SantamaríaAnzhelika VoroshilovaMariana CernicovaAranzazu DuqueMarie DrügeAras BozkurtMarija JevticArfan ShahzadMariko KikutaniAriadna Hernaiz-SanchezMarina EmmanouilArkadiusz JasińskiMarina Leite Mitterer-DaltoéArmand FaganelMarina RezendeArmon TamateaMarina Yurievna PrilutskayaArriel BenisMario A. R. DantasArtemisa R. DoresMarion CoumelArtur C. JaschkeMarisa CorreiaArun SukumarMariusz DzieńkowskiAsad KhanMark HeirigsAshley MandevilleMark R. JohnsonAsier Arcos-AlonsoMark S. FleisherAspasia VlachveiMark W. LuskAtila YildirimMarko SiitonenAtul PandeyMarlene HauptAuditya Purwandini SutartoMarszowski RyszardAugustina Asih RumantiMarta CivljakÀurea Cartanyà-HuesoMarta Regina Cezar-VazAurel BurciuMarta Sainz-GómezAurel NireșteanMarta VelnicAusra DaugirdieneMarta Waliszewska-ProsółAušra KazlauskienėMarta ZnajmieckaAxel SteigerMartin Gomez UllateAyman Abu-RummanMartin Joh. MeyerBalamuralithara BalakrishnanMartin RakusaBarbara J. RyeMartin Sanchez-GomezBarkha YadavMartina MorandoBeata HoffmannMartina OlceseBeata ŁubiankaMarzena LemanowiczBeata SzluzMarzena PodgórskaBeatriz Núñez AnguloMassimo PasquiniBeatriz PereiraMassimo TusconiBegoña UrienMatea Nikolac PerkovicBelem BarbosaMatea Zajc PetranovićBence Péter MarosánMateusz GrajekBibhuti K. SarMateusz JankowskiBidisha RoyMatteo ChiappediBing-Long WangMatteo LandoniBireswar DuttaMatthew J. O’BrienBiswajit MaharathiMatthew J. StainerBo YuMatthew SacchetBobuchi Ken-OpurumMax Carlos Ramírez SotoBogdan GrendaMaxim BakaevBogusława LachowskaMaya MascarenhasBradley LongMayumi KarasawaBratu Mihaela LauraMd Lamiur RaihanBrian BellMeg E. MorrisBrianna Le BusqueMelanie WalkerBrigitte DahmenMerlin Patricia Grueso HinestrozaBruce E. WinstonMicaela Maria ZucchelliBruce WinstonMichael CampbellBruno José Nievas SorianoMichael HastBruno LanfrancoMichael McVeyBruno VendittoMichael SchäferByung Chul ChoiMichael ZanadersC. VijayabanuMichail TheodosiadisCamelia F. OroianMichal ČernýCándido InglésMichal ĎuriníkCarla CanestrariMichał KosakowskiCarla Maria Batista Ferreira PiresMichal VostrýCarlo BieńkowskiMiglė BacevičienėCarlos Armando MendozaMiguel Ángel Albalá GenolCárlos L. Ayán PérezMiguel FilipeCarlos Méndez-MartínezMihai MarianCarlos Mestanza-RamónMihai MusteataCarlos Pérez-GonzálezMikhail F. BorisenkovCarlotta BonMikołaj MagaCarmen Del Pilar Gallardo MontesMilan TomaCarol CarterMiloš HitkaCarolina Blanco FontaoMiloš StankovićCaroline Walker-GleavesMincheol WhangCarrie HowellMing LiuCasimiro Cabrera-AbreuMing-Kuei ShihCatalina MourguesMinh Hieu NguyenCatherine NortonMinjung KimCecilia Zavala-TecuapetlaMinqi YangCésar Merino-SotoMiriam BragaCezar ScarlatMirian Santamaría-PeláezChaang-Iuan HoMirko DuradoniChao GuMirna A. FawazChao HuMiroslav RajterChariklia Tziraki-SegalMiroslava VlčkováCharles Roberto TellesMirosław MatusekChesney CraigMirosława Wielopolska-SzymuraChian Ju JongMo ChenChian-Wen KaoMohamed MohanyChiara MeluzziMohammad MasukujjamanChien-Ming YangMohammad YaminChih-Long LinMohammed HadwanChih-Wen WuMomcilo JankovicChin-An WangMónica Teresa González-RamírezChing Torng LinMónika AndokChin-Hsuan LiuMonika Michałowska-SawczynChintan TrivediMorena MuziChin-Ting LiuMostafa Mohammed Hasan Tawfeek MohammedChris BrownMotoko UeyamaChris RobertsMoudatsou MariaChristian ImbodenMuhammad Ashraf FauziChristine Entleitner-PhlepsMuhammad Azeen AshrafChristoph BorzikowskyMuhammad FareedChristoph VogelsangMuhammad IrfanChristophe DomingosMuhammad Khalid KhanChristy HomMuhammad ShahidChuanyun FuMulazim Hussain AsimChung-Lien PanMurat TezerChung-Tung J. LinMyriam Alvariñas-VillaverdeChung-Wen HungNada MarićCigdem HursenNadine TramowskyCíntia FrançaNarkeesh ArumugamClara Fernández-AlvarezNasheen NurCláudia Maria Ferreira PereiraNasir Muwfaq YounisClaudia StoianNatale MaioranaClaudia TucleaNatalia ManeaClaudiu George BoceanNatalia SuponevaClaudiu MereutaNatalia SzejkoCleo Varianou-MikellidouNathaniel A. ShanokColin J. LewisNebojša PetrovićColleen A. BrennerNeil MartinColleen G. HergottNemanja BerberConcha AntónNenad StevanovicConsolación Gómez-ÍñiguezNgbolua Koto-Te-NyiwaConstantin-Marius ApostoaieNguyễn Văn BiênCorentin J. GoslingNi Wayan MasriCostanza Scaffidi AbbateNicholas ChandlerCristina BravoNicola DöringCristina CiuluvicǎNicola RainisioCristina Lin-LianNicoleta Dorina Racolța-PainaCristina Muñoz Ladrón De GuevaraNicoleta DospinescuCristina Sánchez-MartínezNicoletta CeraCristina SorianoNienke H. Van DokkumCristina TriponNieves ValdésCynthia WhissellNikita MartyushevCzesław NosalNikola KomlenacDaegyu YangNikolaos A. ChrysanthakopoulosDae-Seok KangNila A. WindasariDagmar ČámskáNina EsakiDaisuke MachidaNing-Kuang ChuangDália LiberatoNitiwat WatthanapasDami MoonNoé Macias-SeguraDamian J. RiversNoelia Carbonell BernalDamir PekasNoelia Santamaría-CárdabaDan PăunNolan HigdonDaniel CardosoNoorliza KariaDaniel DemantNorbert SiposDaniel Sanz-MartínNounagnon AgbanglaDaniel SchulzeNyet Moi SiewDaniela Acquadro MaranNyothiri AungDaniela DumbrăveanuOctav Sorin CandelDaniela Soldić FrletaOihane KorresDanielle R. EvangelistaOleg SychevDaniel-Rareș ObadăOlga BrovkinaDaniil ShmatkovOlga MitinaDanuta MakowiecOlga StrizhitskayaDanyelle GreeneOlgierd SwiatkiewiczDaria BylievaOliva Maria Dourado MartinsDariusz DrążkowskiOliver SanderDariusz KałkaOmar DurrahDariusz Wojciech MazurkiewiczOPhir NaveDastan BamwesigyeOrhan KoçakDavid BergeronOscar WoolnoughDavid CokerOurania TremmaDavid Manzano-SánchezPablo A. Cantero-GarlitoDavid MhlangaPablo FaríasDavid TreagustPablo Valdés-BadillaDavid WaynforthPalmira PečiuliauskienėDavide CinoPamayla DarbyshireDawid StormanPamela GiambonaDebasmita BasuPanaoura AretiDeborah GlasoferPaola IannelloDejan MiljenovićPaola ManfrediDespoina PapadopoulouPaolo BennaDiana RusuPaolo CalabresiDimitrios BeliasPaolo RomaDimitrios PanagopoulosPascal IzzicupoDimosthenis KotsopoulosPasquale Fabio ProvenzanoDiva Aliete Dos Santos VieiraPatrícia Silva LúcioDoina PopescuPatrick KwongDolores Lucía Sutil-MartínPatrick PalmieriDominik OlejniczakPatrick TylerDominika WilczyńskaPaul Juinn Bing TanDonald B. GiddonPaul LinksDonald B. StouderPaul McFlynnDonald N. RobersonPaul S. AmieuxDonatas AustysPaula BeneveneDonghwan LeePaula F. Da SilvaDongwon ChoiPaula Fernández GarcíaDongyoup KimPaulo AfonsoDorothee AlfermannPaulo Nuno VicenteDragan PamučarPavlos Ioannis KoktsidisDragisa StanujkicPaweł DębskiDuarte PereiraPaweł PiepioraDulcenombre Gómez-GarrePaweł PrędkiewiczDumitru CiolacPedro Aceituno-AceitunoDushad RamPedro Miguel Alves Ribeiro CorreiaDustin D. WiebePedro Valdivia-MoralE. Begoña Garcia-NavarroPeg SpitzerE. Whitney PollioPei LiuEbba OssiannilssonPeikuan Cor LinEddieson Pasay-AnPerla D. MaldonadoEdgar A. BurnsPerman GochyyevEdina MolnárPeter GlynnEduardo José Fernández RodríguezPeter HervikEduardo Sandoval-ObandoPeter ParryEffrosyni KoutsourakiPeter SmithEfrat RoginskyPeter WakholiEhsan AsadollahiPetra MarkováEl Khalil CherifPetrilla JayaprakashElaine DaviesPhilip PetrovEleanor R. PalserPhilip TsengElena D. BazhanovaPierre GanderElena Jiménez-PérezPilar Munuera GómezElena MacchioniPiotr DzikowskiElena Mainer PardosPiotr KwiatkowskiElena VorobyevaPiotr PoznańskiEleonora TopinoPiotr RatajczakElisa Bizetti PelaiPoh Foong LeeElisabetta Filomena BuonaguroPraveen Kumar NeeliElizabeth BarteltPremnadh Madhava KurupElizabeth EdwardsPrince DonkorElizabeth MorePriyanka SandalElżbieta B. TalikPuiu NistoreanuElżbieta SzymańskaQiyang LiuElzė RudienėQuan-Hoang VuongEmanuele PelosiR. Gabrielle SwabEmem AnwanaRachael PotterEmese Beáta BereiRachel KhanEmil DrápelaRachel SeginerEmilia VassilopoulouRadoslaw B. WalczakEmiliano SironiRafael Almendra-PeguerosEmily Budzynski-SeymourRafael Galán-ArribasEmmanuel TenakwahRafael SaldívarEngin BaysenRafał GerymskiEnrico BocciolesiRafał JureckiEnriqueta Canseco-GonzálezRafat GhanamahEric AgboliRahshida AtkinsErin CrawRainer LeonhartErin GrinshteynRajat Emanuel SinghErinn HawkinsRajkishor KumarErmanno TortiaRamazan YirciErnesto Leon-CastroRamesh KumarErnesto RosarioRamey MooreErsan ArslanRamona HenterErtuğrul ŞahinRaquel CostaEster NavarroRasa JankauskieneEugenio De GregorioRasa PranskunieneEun-Jung LeeRasa SmaliukienėEvelin WitrukRaul NavarroEvelina KristanovaRaúl Quevedo-BlascoEvgenia SitnikovaRazvan DinaEwa FiedorowiczRebekah WadaEwa HąciaRemco S. MannakEwa JaskaRemus CretanEwa JastezębskaRenata GambinoEwelina Pogorzelska-NowickaRenata Jadresin MilicF. Javier Del RíoRenáta MachováFabian BehrendtRenata ŠkrbićFábio PyRenato CostaFabio SantacaterinaRicardo Costa ClimentFabio SereniRicardo José Pinheiro MoraisFabrizio Terenzio GizziRichard L. WolfelFahimeh GolbabaeiRichard MorrisFaizah Safina BakrinRima KregždytėFanli JiaRiquelme Marín AntonioFanny Forsberg LundellRishabh C. ChoudharyFátima Chacón-BorregoRita PeresFederica SgarbiRita Tavares De SousaFederico InnocentiRita Yi Man LiFederico ZappaterraRiwanti EstiasariFernanda MatiasRiyan Van Den BornFernando Austria-CorralesRobert A. MacphersonFernando CarvalhoRobert Emmett KellyFernando SantosRobert James CrammondFernando TavaresRobert M. CariniFesto KayimaRoberta SalaFezile OzdamliRoberto Soto VarelaFiorella Pia SalvatoreRoberto VagnettiFiroz AkhterRocío Llamas-RamosFlorian HeilmannRocío Valderrama-HernándezFlorian PochsteinRodney DuffettFlorian SchmitzRomana Korez VideFlorin LeuciucRona HartFolajinmi OluwasinaRonald HübnerFrances L. M. SmithRondy YuFrancesca CiampaRonen HershmanFrancesca Giulia MagnaniRongxu QiuFrancesca VitaleRosa-Eva Valle-FlórezFrancesco DemariaRosanna Mestre PérezFrancesco Domenico Di BlasiRoy AloniFrancesco Perono CacciafocoRuben Lopez-BuenoFrancisco Javier Flor MontalvoRubén Rivas-de-RocaFrancisco Javier García-PrietoRubén San-SegundoFrancisco Jose Garcia-MoroRubén TriguerosFrancisco MonteiroRubinia Celeste BonfantiFrancisco PerezRupesh Kumar ChikaraFrancisco SampaioRu-Si ChenFranziska LautenbachRuslan KravetsFriederike PaetzRuss MarionG. D. M. N. SamaradiwakaraRuxandra BejinaruGabriela BoldureanuRyohei NakatsuGabriela TopaRyuichi OhtaGakuji KumagaiS. M. Sohel MahmudGang GuoS. SasikalaGeoffrey Harvey TanakinjalS. Zaung NauGeorg JuckelSabahudin HadžialićGeorge FeinSabina NeculaGeorge LazaroiuSabrina Oktoria SihombingGeorgi PanovSaeid Norouzian-MalekiGeorgia RowleySaima ShafiqGeorgina FauraSaleh BajabaGerald AranoffSalvatore MonacoGermán Zárate-SándezSamayan NarayanamoorthyGermano RossiSamia ElseginyGessynger Morais-SilvaSampath Kumar VenkatacharyGheorghița NistorSamuel P. LeónGhulam AbidSanar MuhyaddinGianluca EspositoSandra Mrvica MadaracGianluca SerafiniSandro Fernandes Da SilvaGilberto GalindoSang Soo KimGilberto Gonzalez-ParraSang-Dol KimGintarė VaznonienėSangyong JungGiovana Goretti Feijó De AlmeidaSanna StrothGiovanna CampaniSara Domínguez-LloriaGiovanna FurneriSara LunaGiovanni CangelosiSara Owczarczak-GarsteckaGiulio GabrieliSarah Hian May ChanGiuseppe VaroneSarah SteimelGiusy Danila ValentiSaral DesaiGloria GuidettiSarfaraz Hashemkhani ZolfaniGonzalo Díaz-MenesesSatoshi UchidaGoran SoderlundSatwant KumarGordon BarbaraSawsan M. A. AbuhamdahGorka VuletićSebastian WeirichGovind KrishnamoorthySebastiano FaroGrace A. GomashieSeoyoon HeoGrzegorz DrozdowskiSepehr AlizadehsalehiGrzegorz PolokSergei IgumnovGuadalupe Molina TorresSergej GričarGudrun Vande WalleSergey DydykinGuilhermina Lobato MirandaSergey Valentinovich BorisovGuillermo Gómez-DelgadoSergio Calonge-PascualGuillermo PlazaSergio López-GarcíaGuillermo Sanahuja-PerisSergio Ochoa JiménezGunilla BohlinSerhii LupenkoGunnar MauShafique Ur RehmanGuowen ShangShaina PonceGurvinder KaurShamik ShahGustavo Vieira De OliveiraShang-Pao YehGuzide SenelSharlene GillGyörgy Iván NeszmélyiSharon JaleneHady KeitaSharon K. HunterHajnal IstvánSharon MartinoHalime AbayShawna Malvini ReddenHalyna MishchukShehla RazviHani Amir AouissiShifang TangHanna LiberskaShihai LiHannah JonesShin’ya NagasawaHannah ThorneShin-Cheng YehHao MaShirley HillHao ZhangSho TakahashiHarriet BradleyShu-Ching MaHarsh V. VermaShulamit PinchoverHarukaze YatsugiSilja MartikainenHassan BanarueeSilvana Mabel Nuñez-FaddaHaywantee RamkissoonSilvana Maria Russo WatsonHeather CarmackSilvia CataldiHéctor Hugo Pérez-VillarrealSílvia LopesHeejung ParkSilvia Morales ChaineHeidi HillmanSilvio IontaHeidi L. DempseySimona Catalina StefanHélder RaposoSimona GoiaHera AntonopoulouSimona RaimoHervé Laborde-CastérotSimona VinereanHilary De ShongSimone BattagliaHina MunirSimone BelliHiroko OchiaiSimone Di PlinioHo Hon LeungSimone DonatiHon Lon TamSimote FoliakiHongfang WangSina HafiziHoupu LiSiniša OpićHugo Machado SanchezSiwei ZhangHumberto Javier Rodríguez HernándezSiyuan HuangHye Eun LeeSławomir GawrońskiHyoung Chul ShinSławomir ZapłataHyoung-Kil KangSofia GomesHyun Jung ParkSofia PapavlasopoulouHyung Rok WooSonia Brito-CostaIbrahim ArpaciSónia Maria Martins CaridadeIbrahim M. SayedSonika SinghIda Claudia PanettaSoon Singh BikarIgnacio Ramos-VidalSorush NiknamianIgor MandelSoumitra DasIgor RyabovŠpela SelakIlaria BertocchiStacy WassellIlaria SettiStefania ToselliIlma MufidahStefano DastoliIlona Rubi-FessenStefano RuggieriIlse ScheerlinckStella TsotsiIlze BeitaneStepan FeduniwImran AslanStephanie KershawIna KayserStephanie Wing LeeIndika Neluwa-LiyanageSu ZhangInés Llamas-RamosSue SoanInmaculada Rodríguez-CunillSukeun JeongIoana-Crina Pop-CohuţSunitha ZechariahIon PopaSuravajhala PrashanthIonut LuchianSusan RasmussenIqram HussainSusana BernardinoIrene TilikidouSusana BorgesIrida HotiSusana Martínez-RodríguezIrina Eduardovna SokolovskayaSusana PaixãoIrineu De Brito JuniorSusana SilvaIrving PaputunganSusana Sofia MiguelIsaak PapadopoulosSusanne BrouwerIsabel Corrales GutiérrezSusanne M. Van Der VeenIsabel Rodrigo MartínSuzana DemyenIsac Florin LucianSyafrizal Helmi SitumorangIsilda RibeiroSyaharizatul Noorizwan MuktarIsmael Cristofer BaierleSydney AtenIulia MuresanSylvia MignonIva BužančićSylvia TerbeckIvana OleckáSzabolcs SzámadóIvano CaselliTabussam TufailIvette Margarita Espinoza-DíazTae Im KimIza GigauriTahereh MaghsoudiIzabela ParowiczTakaaki AratakeIzabela StalończykTakahiro NemotoIzhak SchnellTakashi G. SatoJaana SimolaTakuma InagawaJacobo Cano EscoriazaTamar BasishviliJacopo LisoniTamara BozovicJaehee HanTamara MohorićJagdeep RahulTamás DócziJagdish KhubchandaniTânia LucciJaime NavarreteTanja BukovčanJaime Rivera CaminoTao WangJaime Vásquez-GómezTatiana RameyJairo L. VanegasTatjana PortnovaJakub KołodziejczykTeerasak JindabotJakub RajčániTetyana PimonenkoJakub SwachaTharshanah ThayabaranathanJames C. SimeonTheam Foo NgJames DillardTheodoros FouskasJames O. FinckenauerThiago VianaJames W. SonneThiago VieiraJames WilliamsThierry MeyerJamie MarsdenThomas AkintayoJamiu Adetola OdugbesanThomas D. BuckleyJan K. WachterThomas J. MeyerJán KnapíkThomas Rhys EvansJan P. RöerTibor ZsigmondJana FedtkeTiffany J. Ríos-FullerJanet A. FooteTiija RintaJarosław UglisTimo AiraksinenJason James TurnerTimothy M. EschleJavier Almela-BaezaTimothy T. SelfJavier Esparza-ReigTina CoffeltJavier TarangoTina KubrakJaynie RanceTinakon WongpakaranJean Christophe GigerTine K. GrimholtJeanette KlinkTing Kin NgJean-Francois DaoustTingting ZhangJeanne SlatteryTodd L. MatthewsJeanne WenosTom SherwoodJeffrey Hugh GambleTomas KrulickyJeffrey SchweitzerTomás TeodoroJehad AliTomasz GigolJelena MušanovićTomasz WarchołJennifer BriteTomiko YonedaJennifer R. WarrenTomina Gabriela SăveanuJennifer TaberTommaso LomonacoJenny HuangTomoko NishimuraJeonggyu HuhTonglin JiangJerzy RomaszkoTracy DonachieJessica Mayra FerreiraUlrike GelbmannJessica WelchUmut ÖzkayaJessie MartinUnnur OttarsdottirJesús Miranda PáezUrsula MartinezJesús-Nicasio García-SánchezUtpal NandiJiafu SuVálber César Cavalcanti RozaJian-Hong YeValentina BaglioniJiaqi LiValentina ChkoniyaJie YinValentina Maria MerlinoJing TengValentina NiccolaiJinjin LuValentina RusuJiri PospisilValerie CainesJitse P. Van DijkValeriia DemarevaJoana Silva RibeiroValerio RicciJoan-Francesc Fondevila-GascónVanja KopilašJoanna ŁukasikVassilios PanoutsakopoulosJoanna PfingsthornVelga SudrabaJoannes ChliaoutakisVeli-Matti VesterinenJoão Carlos MacedoVera BrenčičJoão Pedro BaptistaVeronica CarnicelliJoaquín NavajasVerónica Mäki-MarttunenJodye SelcoVeronika KotradyováJoel ParthemoreVictor CepoiJohanna TakácsVijay VictorJohannes BlumViji VijayanJohannes Kopf-BeckViktor FredrichJohannes T. DoerflingerVincent PonsJohn Amson CapitmanVincenzo De LucaJohn EastwoodVincenzo Nunzio ScalcioneJohn P. WilsonViorel MihailaJolanta ChmielowiecVirgilio López-MoralesJolanta MackowiczVirginia RecchiaJolanta PułkaVirtudes Pérez-JoverJolanta StrzeleckaVivek KumarJonatas Rafael De OliveiraVladislava LendzhovaJoon W. ShimVsevolod V. KonstantinovJordan PierceWai-Sum LeeJordi Franch-ParellaWaldemar CudnyJorge Amate-RomeraWang-Kin Oscar ChiuJorge Calvillo-ArbizuWarawoot ChuangchaiJorge García-VillanuevaWee Meng Eric LeeJorge Humberto Palmeira RamosWei LiuJorge MachadoWei-Ting HsuJorge Moya HiguerasWenceslao PéñateJorge Rodríguez-ArceWendy PackmanJose A. BragadaWeng Marc LimJosé Belda MedinaWeon-Young LeeJosé Carlos Vázquez-ParraWesam Al MadhounJosé Castro OliveiraWiesław FideckiJosé J. Martínez-MagañaWillard MunyokaJosé MaganoWilliam CheungJose Manuel Saiz-AlvarezWojciech CzerskiJosé María León-RubioWojciech StykJosé MartinsWonkyong Beth LeeJosef BotlikWren StevensJosé-María FigueredoXia HaoJoseph BaumgardenXiao HanJosko SodaXiao YangJuan Aníbal González-RiveraXiaojun DingJuan Carlos Checa-OlmosXiaopeng JiJuan Carlos Infante-MoroXiaoshan GordyJuan De Dios Benítez SilleroXiaoshan LiJuan González-MartínezXin-Long XuJuan González-PascualXinxing WangJuan M. Santos GagoXuan HaJuan Miguel Benito-OstolazaXuan MaJuan Pedro Martínez RamónXuemei ZhangJuan-Francisco Álvarez-HerreroXueying WangJuan-Ramón Moreno-VeraYaara Endevelt-ShapiraJudit Orgoványi-GajdosYan MuJudyta KabusYancy FerrerJueun KimYawei LiJui-Hsiang LeeYe ChenJulian McDougallYesim SireliJulie FitnessYi -Fang HsiehJulie J. KeysorYi-Fang LeeJulie ScorahYing-Kai LiaoJumadil SaputraYolanda Marcén-RománJun LiYomna MahmoudJunfeng YangYongli LiJunhyung KimYoshihisa Hirakawa‪Junia Nogueira De BritoYoshinori MoriyamaJustin R. FeeneyYuane JiaJustyna MrózYuanyuan LanJuvenal Rodríguez-ReséndizYue ZhangJyh-Jeng WuYueyun ZhangKah Hui WongYufeng LaiKa-Huen YipYuka KotozakiKai LiuYunjia ChiKamel FantazyYuri KopylovKanchan MarcusYusuke KajiwaraKaren LillerYutaka OwariKarina Stasiuk-KrajewskaqYuyang DongKarol DąbrowskiYvonne ThaiKarol KonaszewskiZacharias PapadakisKarolina Eszter KovacsZahid YousafKarolina KrysinskaZahra PanjaliKasper SipowiczŽaneta GerhátováKassegn Berhanu MeleseZbârcea Cristina ElenaKatarzyna JachZekâi ŞenKatarzyna PawlikowskaZeki Atıl BulutKatarzyna Pawlikowska-ŁagódŽeljko PavićKatarzyna TomaszekZhengdong ZhaoKatharina S. SchuhmannZia BashirKatherine OgurtsovaZihe GaoKathi R. TrawverZoltan BaracskaiKathy Merlock JacksonZsófia KeneseiKatie Fitton DaviesZsuzsanna KöviKatie PageZuzana LušňákováKatinka BacskaiZuzana StrakováKavitha HaldoraiZuzana VišňovcováKayode Kolawole EluwoleZvonimir Šatalić

